# Growing with our authors: Welcoming a new Deputy Editor and three new Associate Editors to the *Journal of Experimental Orthopaedics*

**DOI:** 10.1002/jeo2.70879

**Published:** 2026-08-06

**Authors:** Stefano Zaffagnini

**Affiliations:** ^1^ Clinica Ortopedica e Traumatologica II IRCCS Istituto Ortopedico Rizzoli Bologna Italy; ^2^ Dipartimento di Scienze Biomediche e Neuromotorie (DIBINEM) University of Bologna Bologna Italy

**Keywords:** *Journal of Experimental Orthopaedics* (*JEO*), orthopaedic research, scientific publishing

**Abbreviations**
ESSKA: European Society of Sports Traumatology, Knee Surgery and Arthroscopy
*JEO*
: *Journal of Experimental Orthopaedics*

Every manuscript that reaches the *Journal of Experimental Orthopaedics* (*JEO*) begins its life in the hands of an editor. Long before a paper is cited, discussed at a congress or translated into a change in clinical practice, someone has read it carefully, asked whether the question was worth asking, whether the methods could answer it, and whether the conclusions were honest. That quiet, largely invisible work is the real infrastructure of a scientific journal, and it is the reason why the composition of an editorial board is never a purely administrative matter.

*JEO* has grown considerably over the past few years. As the official experimental and translational journal of the European Society of Sports Traumatology, Knee Surgery and Arthroscopy (ESSKA), and as a fully open‐access publication, we have seen a steady increase in submissions, an expanding geographical footprint, and an increasingly diverse portfolio of study designs from bench‐side biomechanics and animal models to registry analyses, systematic reviews with robust methodology, and early clinical translation. Growth of this kind is welcome, but it is also demanding. It requires editors who combine scientific rigour with speed, methodological literacy with clinical judgement, and perhaps most importantly, a genuine willingness to invest time in other people's work.

It is therefore with great pleasure that I announce the promotion of Luca Macchiarola to Deputy Editor, and the appointment of George Mihai Avram, Lika Dzidzishvili and Theodorakys Marín Fermín as Associate Editors of *JEO*. All four have already served the journal as reviewers, as members of the Editorial Board, and in several cases as award‐winning contributors to its scientific content. Their promotion is not the beginning of their relationship with *JEO*; it is the formalization of a commitment they have already demonstrated.


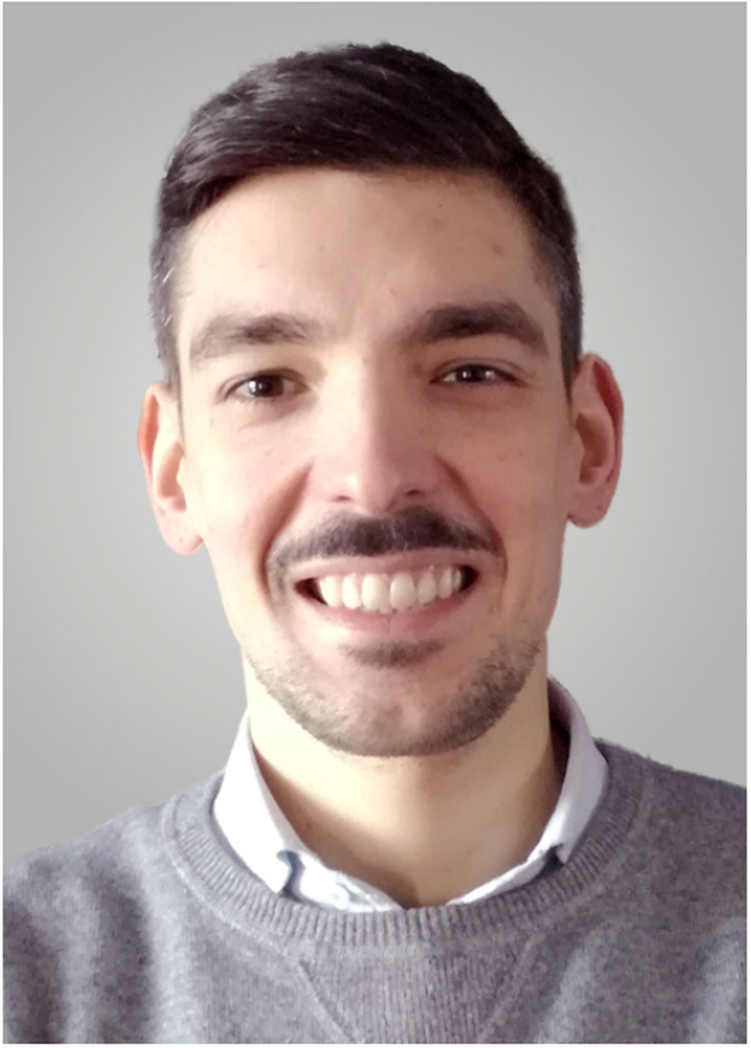


**Luca Macchiarola—Deputy Editor**

Luca Macchiarola brings to the role of Deputy Editor an unusual combination of clinical, scientific and editorial maturity. Trained in Orthopaedics and Traumatology at the Istituto Ortopedico Rizzoli in Bologna and now an Attending Physician at Istituto di Ricovero e Cura a Carattere Scientifico (IRCCS) Casa Sollievo della Sofferenza in San Giovanni Rotondo, he completed a PhD in Translational Medicine at the University of Foggia and obtained the Italian National Scientific Qualification as Associate Professor. His international training, including a clinical research fellowship at the Centre Hospitalier de Luxembourg and a surgical fellowship in the Sports Surgery Department of Aspetar, Doha, has given him a first‐hand understanding of how research questions are framed differently across systems and cultures.

Scientifically, his contributions span anterior cruciate ligament reconstruction and revision surgery, extra‐articular lateral procedures, meniscal allograft transplantation and collagen meniscus implants, in vivo kinematic assessment with navigation systems, paediatric knee injury and the cross‐cultural validation of outcome measures. This is a body of work built on long‐term follow‐up and objective measurement rather than on short‐term novelty, precisely the ethos *JEO* seeks to promote.

Equally relevant is his editorial track record: He has reviewed for a long list of journals, was awarded *JEO* Best Reviewer in 2019, and has served as a certified reviewer mentor. Reviewing is a skill that can be taught, and Luca is one of the people who teaches it. In his new role as Deputy Editor, he will support the strategic and day‐to‐day management of the journal, with particular attention to editorial quality, turnaround times and the mentoring of our reviewer pool.


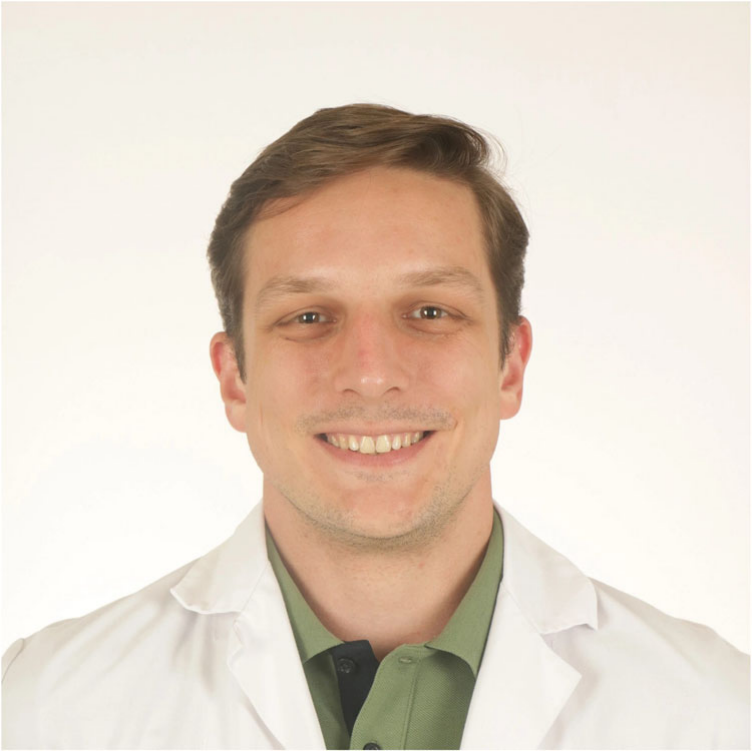


**George Mihai Avram—Associate Editor**

George Mihai Avram represents the generation of surgeon‐scientists that *JEO* explicitly wants to attract. Currently completing his orthopaedic residency in Bucharest and a research member of Michael T. Hirschmann's group at the University of Basel, he has already built a substantial portfolio in personalized and robotic‐assisted arthroplasty, knee phenotyping and alignment classification, alongside work in shoulder sports medicine.

What distinguishes him, however, is his investment in research methodology and scientific education. He chairs the Scientific Committee of the Personalized Arthroplasty Society and the Education Committee of the Romanian Society of Sports Traumatology and Arthroscopy, where he co‐founded an educational platform and has run a series of webinars teaching residents and medical students how to write abstracts and conduct systematic reviews. He was named *JEO* Reviewer of the Year in 2023, an award I had the pleasure of presenting personally and has since received similar recognition elsewhere. His appointment strengthens our capacity to handle methodological and evidence‐synthesis submissions, an area where the volume of submissions has grown faster than the quality, and where firm, constructive editorial guidance is most needed.


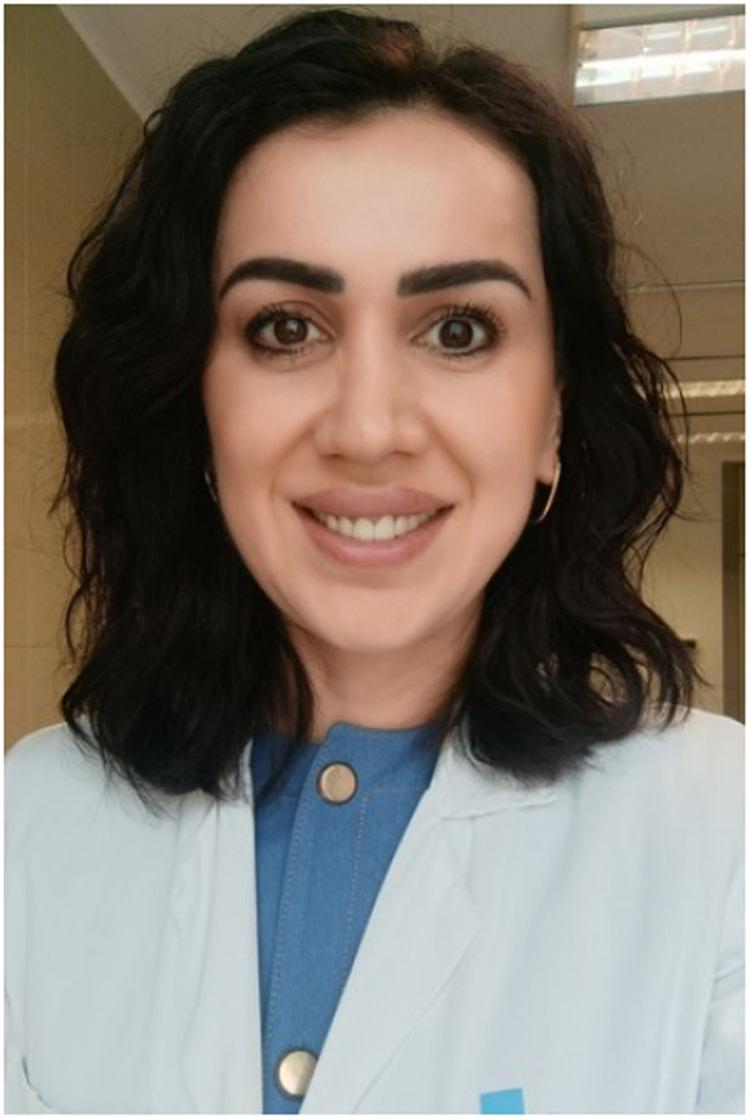


**Lika Dzidzishvili—Associate Editor**

Lika Dzidzishvili joins the editorial team with one of the strongest basic and translational science profiles among our reviewers. An attending orthopaedic surgeon at Hospital Universitari Germans Trias i Pujol in Barcelona, with a PhD cum laude from the Universidad Autónoma de Madrid and a research fellowship at Rush University Medical Center in Chicago, she has focused on meniscal root pathology from in vivo experimental models of post‐traumatic osteoarthritis and histological analysis of human menisci, to biomechanical meta‐analyses and clinical outcome studies as well as on shoulder instability.

Her work has been recognized repeatedly at the highest level, including the International Society of Arthroscopy, Knee Surgery and Orthopaedic Sports Medicine (ISAKOS) Albert Trillat Young Investigator Award, the ESSKA Porto Award for Innovation in Arthroscopy, an ESSKA Basic Scientist Abstract Award, and closer to home, the *JEO* Young Researcher Award in Basic Science. She also completed the *JEO* Editorial and Research Fellowship in Bologna and serves on the ESSKA Basic Science Committee and two ISAKOS committees. If *JEO* is to remain the natural home for rigorous experimental orthopaedic research, we need editors who can evaluate an animal model, a histological grading system or a biomechanical testing protocol with the same confidence with which they read a clinical cohort. Lika is exactly such an editor.


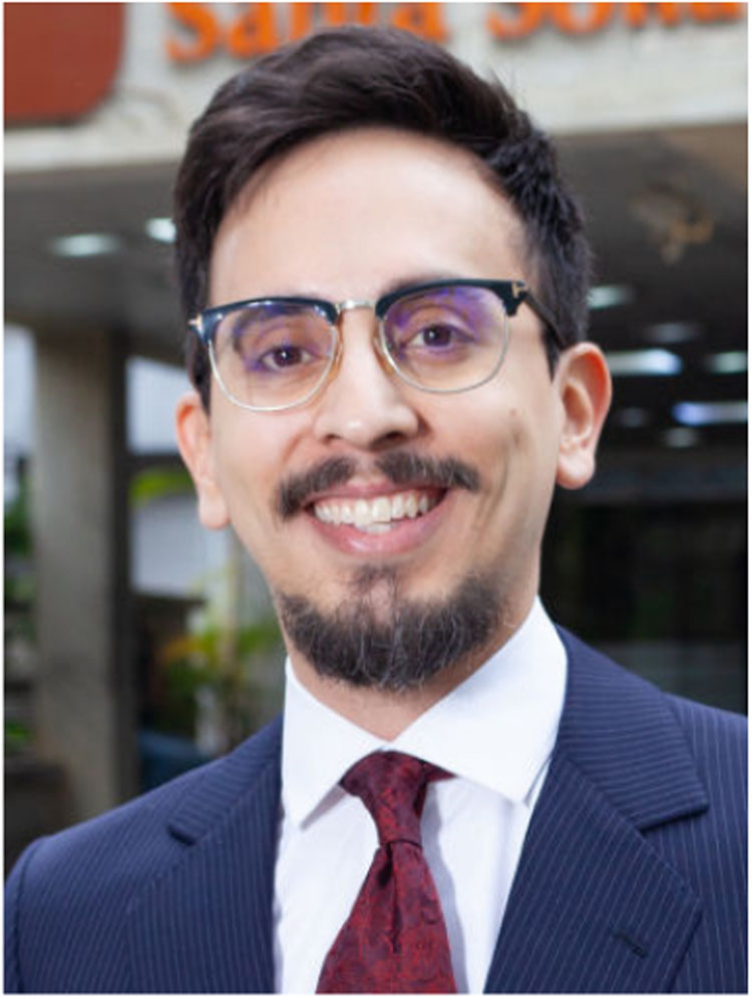


**Theodorakys Marín Fermín—Associate Editor**

Theodorakys Marín Fermín brings to the board a global perspective that *JEO*, as an open‐access journal, has a particular duty to cultivate. An orthopaedic surgeon based in Caracas, with fellowship training in sports medicine and arthroscopy in Thessaloniki and in sports surgery and clinical orthobiologics at Aspetar, he is the author of more than sixty manuscripts and book chapters, concentrated in orthobiologics, cartilage repair, joint preservation and sports injury.

His work on platelet‐rich plasma, including critical appraisals of preparation techniques, reporting standards and safety, addresses one of the areas where the gap between commercial enthusiasm and reliable evidence remains widest, and where a journal such as ours has a responsibility to insist on transparency and reproducibility. Beyond his scientific output, he serves as Patient Registry Manager for the International Cartilage Regeneration & Joint Preservation Society and sits on several ISAKOS committees, and he has been a persistent advocate for overcoming language and resource barriers in orthopaedic education and publication. He received the *JEO* Best Reviewer award in 2022, and his appointment reflects both his editorial reliability and our determination to be a journal that is genuinely international in practice, not only in ambition.

## WHAT THIS MEANS FOR *JEO*

These four appointments are not merely a refresh of the masthead; they express a strategic direction.

First, methodological rigour. As submission volumes rise, the differentiating factor for a journal is no longer how much it publishes but what it declines to publish, and how well it explains why. Our expanded editorial team has been chosen for its ability to interrogate statistical, biomechanical and experimental methods and to give authors feedback that improves their work rather than merely judging it.

Second, the full translational chain. *JEO* exists precisely to cover the space between the laboratory and the operating room: basic science, biomechanics, animal and cadaveric models, imaging, registry data, early clinical translation. The profiles above experimental models and histology, kinematics and navigation, phenotyping and personalization, orthobiologics and cartilage map deliberately onto that chain.

Third, mentorship and the next generation. Three of our four new editors are actively engaged in teaching young colleagues how to review, how to write and how to design a study. Peer review is a skill under strain across all of medicine; a journal that does not train its reviewers will not have any in 10 years' time. Expect to see further initiatives from *JEO* in this direction, building on our editorial and research fellowship programme.

Fourth, genuine internationality. Italy, Romania, Spain and Georgia, Venezuela and Qatar, Switzerland and the United States: The paths of our new editors cross most of the scientific world. Open access is only meaningful if the door opens in both directions, if authors from every region can publish with us, and readers from every region can use what we publish.

To Luca, George, Lika and Theodorakys: Thank you for accepting these roles, and welcome. To our authors and reviewers: you are the reason the journal exists, and these appointments are made with your work in mind. And to those considering where to submit their next experimental or translational study, we look forward to reading it.

**Stefano Zaffagnini** *Editor‐in‐Chief, Journal of Experimental Orthopaedics*

## CONFLICT OF INTEREST STATEMENT

The author declares no conflicts of interest.

